# Micronutrient Intakes among Women of Reproductive Age in Vietnam

**DOI:** 10.1371/journal.pone.0089504

**Published:** 2014-02-21

**Authors:** Phuong H. Nguyen, Hieu Nguyen, Ines Gonzalez-Casanova, Erika Copeland, Garrett Strizich, Alyssa Lowe, Hoa Pham, Truong V. Truong, Son Nguyen, Reynaldo Martorell, Usha Ramakrishnan

**Affiliations:** 1 Thai Nguyen University of Pharmacy and Medicine, Thai Nguyen, Vietnam; 2 International Food Policy Research Institute, Hanoi, Vietnam; 3 Hubert Department of Global Health, Rollins School of Public Health, Emory University, Atlanta, Georgia, United States of America; 4 Mailman School of Public Health, Columbia University, New York, New York, United States of America; University of Pennsylvania, United States of America

## Abstract

**Background:**

Micronutrient deficiencies are a public health concern worldwide negatively affecting maternal and child health outcomes. The primary underlying causes of micronutrient deficiencies are insufficient intake and poor bioavailability of micronutrients. However, reliable data on micronutrient intakes are sparse. The objectives of this study were to identify the key local food sources providing the majority of micronutrients and assess the adequacy and determinants of micronutrient intakes.

**Methods:**

The study used data from a survey of 4,983 rural women of reproductive age (WRA) participating in a preconception micronutrient supplementation trial in Vietnam. Micronutrient intakes were assessed using a validated 107-item semi-quantitative food-frequency questionnaire. Multivariate linear and logistic regression analyses were used to examine the association between socioeconomic status and micronutrient intakes.

**Results:**

Starchy staples were the main source of iron and zinc (37% and 54%, respectively) with only a small proportion from meat (10% and 18%, respectively). The primary source of folate and vitamin A were vegetables; vitamin B12 came from meat and eggs. The proportion of the population with intakes below the estimated average requirement was 25% for iron, 16% for zinc, 54% for folate, 64% for vitamin B12 and 27% for vitamin A. Socioeconomic status was the main determinant of micronutrient intakes. WRA in the highest quintile consumed 26% more iron, 19% more zinc, 36% more folate, 82% more vitamin B12 and 47% more vitamin A compared to those in the lowest quintile. Women in the upper quintiles of SES were more likely to obtain nutrients from more nutritious and higher bioavailable foods than those in the lowest quintile.

**Conclusions:**

Underprivileged women were at increased risk for insufficient micronutrient intakes due to poor diet quality. Targeted efforts to promote the consumption of local nutrient rich foods along with educational programs and social development are needed.

## Introduction

Micronutrient deficiencies are a significant public health problem in developing countries, particularly in Southeast Asia [1], [2]. Micronutrients include vitamins and minerals that aid the body in the production of hormones, enzymes, and other substances that are critical to the normal growth, development, and functionality of the body [3]. The World Health Organization contends that the micronutrients iron, iodine, zinc, folic acid, and vitamin A are among the most critical for maternal and child health [3]. However, deficiencies of these same nutrients are the most common among women of reproductive age (WRA) and are associated with increased risk of adverse consequences such as anemia during pregnancy and maternal mortality, pre-term birth and/or low birth weight infants [4], birth defects [3], increased mortality and suboptimal health and cognitive development of the offspring [5].

Micronutrient deficiencies are critical maternal and child health concerns in Vietnam [6]. According to the latest national nutrition survey, anemia affects 28.8% of non-pregnant women, 36.5% of pregnant women and 29.2% of children under 5 years of age [7]. The prevalence of vitamin A deficiency among children under five is 14.2% and is highest among children under 12 months [7]. Zinc deficiency was reported in 67% and vitamin B12 deficiency was 12% among WRA [8].

The primary underlying causes of micronutrient deficiencies are insufficient intake and poor bioavailability of micronutrients. However reliable data on micronutrient intakes are sparse. A 2004 food consumption assessment revealed that energy and macronutrient intake in Vietnam varied by socio-economic status and demographic characteristics including place of residence, wealth quintile and education level [9]. We found two recent studies that evaluated micronutrient intakes in Vietnam; one examined relationships between SES and micronutrient intakes [10] while the other focused on identifying suitable food vehicles for fortification [11]. These studies however estimated nutrient intakes using household level data rather than individual consumption which may vary within households and also did not examine how consumption varied by vulnerable groups, such as rural communities or ethnic minorities.

This paper fills the gap in the literature by addressing three objectives: 1) to assess the adequacy of micronutrient intakes among WRA mainly in rural communities in northern Vietnam; 2) to identify the key local food sources providing the majority of micronutrients; and 3) to examine the influence of household SES on individual micronutrient intakes and their food sources. Knowledge of the food sources being consumed by WRA and of the micronutrient deficiencies they suffer will help design appropriate education and counseling materials, and stratification by socioeconomic status will allow us to make recommendations for targeted nutrition interventions.

## Methods

### Data Source and Study Population

This study used baseline data that were collected as part of a large randomized control preconceptional micronutrient supplementation trial (PRECONCEPT) aimed at improving maternal and infant health [12]. The baseline survey was conducted between November 2011 and April 2012 in four of the nine districts of Thai Nguyen province, in northern Viet Nam. These districts have high proportions of ethnic minority women whose main occupation is farming; the key crops are rice and tea, and the majority of foods consumed are locally produced. A list of all WRA was obtained from Commune health center (CHC) for each commune. The village health workers (VHW) visited the homes of all women on the list who were married and not pregnant at the time to inquire about their plans for pregnancy in the upcoming year. All women intending to get pregnant within the next year were invited to participate in the study. There were no differences in basic baseline characteristics among those who participated and those who did not (result not shown).

The study received ethical approval from the Institutional Review Board from the Institute of Social and Medical Studies in Vietnam and from Emory University. Written informed consent was obtained from all participants before taking part in the study.

### Measures

#### Vitamin and mineral intakes

Trained interviewers collected information on dietary intakes using a semi-quantitative food frequency questionnaire (FFQ) that was developed and validated by Vietnam’s National Institute of Nutrition (NIN) [13]. The FFQ includes a list of 107 common food and beverage items. Participants were asked to report how frequently they had consumed each specific food over the past three months. Average portion sizes were determined using a standardized collection of commonly used tableware, and the average number of servings per meal was obtained.

Vitamin and mineral intakes were estimated from FFQ data and Vietnamese food composition tables [14]. Complex foods not included in the database were broken down into individual component ingredients taken from a common Vietnamese recipe book [15], and nutrient contents were calculated. In order to determine which food groups provide specific nutrients to different socio-economic groups, the 107 food items were organized into 10 groups: 1) grains, roots and tubers; 2) legumes and nuts; 3) vegetables; 4) fruits; 5) oil, lard and butter; 6) meat, organs and meat products; 7) fish and shellfish; 8) eggs; 9) milk and other dairy products; and 10) sugar, sweets, condiments, and beverages. Micronutrient intakes from each food group were calculated. Since the Vietnamese diet is predominantly cereal-based with a low consumption of animal source foods, we assumed low bioavailability to define the recommended dietary intake for iron (12% bioavailability instead of 18%, as in a Western diet) and zinc intakes. Intakes below the estimated average requirement (EAR) were defined as inadequate [16].

#### Independent variables

Socio-economic status was assessed using a structured questionnaire that included questions related to house and land ownership, housing quality (e.g., house construction materials), access to services (water, electricity, gas and sanitation services) and household assets (various durable goods, productive assets, animals and livestock). The SES index was constructed by principal components analysis [17], [18], and was categorized into quintiles. Socio-demographic characteristics, including age, education level, ethnicity and occupation were collected by structured interview. Education was divided into 4 categories based on highest grade level completed. Ethnicity was divided into 2 categories: Kinh majority and all minority groups. Occupation was categorized as farmer or other. Children (<18 years of age) and elderly (≥65 years of age) were classified as dependents.

### Statistical Analyses

The Kolmogorov-Smirnov test was used to assess normality. Log transformation was used to normalize the variables that were not normally distributed. Descriptive analyses were used to report demographic and socioeconomic characteristics of the study sample. Median intakes and interquartile ranges were calculated for each nutrient. Bivariate analysis was performed to examine differences in nutrient intakes by quintiles of SES using ANOVA test for continuous variables and Chi-square test for categorical variables.

Multivariate analysis was done for five micronutrients, namely iron, zinc, folate, vitamin B12 and vitamin A. We used multivariate linear regression models to examine the association between SES and micronutrient intakes, adjusting for potential confounding factors (age, education, ethnicity, occupation, energy intake, clustering effects at the commune level and timing of data collection). Differences in nutrient intakes by quintiles of SES were expressed as percentages rather than absolute values due to the use of log transformation. We used multivariate logistic regression models to calculate adjusted odds ratios (95% confidence interval) for inadequate intakes of the selected micronutrients. Finally, we compared the relative contribution of different food groups to micronutrient intakes by quintiles of SES. All statistical tests were 2-tailed and differences were considered significant at P<0.05. SAS software version 9.2 was used for statistical analysis [19].

## Results

### Descriptive Characteristics of Study Sample

Complete data for dietary intakes and socio-demographic characteristics were available for 4,983 out of 5011 WRA who participated in the baseline survey. The mean age (±SD) of study participants was 26.2±4.6 y. Farmers represented 80.6% of the sample. More than half the participants were of the Kinh ethnic group (50.5%). The minority group included Tay (17.4%), Nung (12.7%), Dao (5.4%), San Chi (7.0%), and San Diu (2.2%). More than half of the participants (54.7%) completed secondary school; 25.1% completed high school; 12.0% completed at least one year of higher education and only 8.2% had less than a primary school education. The large majority, 74.2% of respondents, lived in households with one dependent, while 7.8% had zero dependents and 18.1% had two or more dependents. The mean body mass index (BMI) (±SD) for the study sample was 19.5±1.9 and the prevalence of underweight (BMI<18.5 kg/m^2^) and overweight (BMI≥23 kg/m^2^) were 31.7% and 5%, respectively. Mean hemoglobin concentration was 13.0±1.4 g/dL and anemia was present in 19.7%.

### Key Micronutrient Intakes

**Figure 1:**
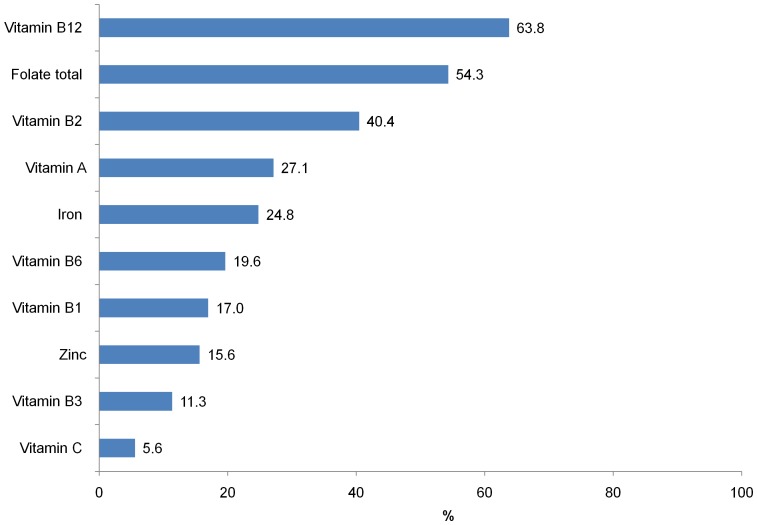
Prevalence of inadequate intakes (below the estimated average requirement) of selected micronutrients among WRA in rural communities in northern Vietnam.

The dietary intakes for the micronutrients of interest are shown in Table 1. The overall median intakes of several micronutrients are above the EAR but average daily intakes were much lower than the EAR for iron, folate and vitamin B12. The proportions intakes below the EAR were 24.8% for iron, 15.6% for zinc, 54.3% for folate, 63.8% for vitamin B12 and 27.1% for vitamin A (Figure 1). The level of insufficient intake ranged from 11–40% for different B vitamins. Examination of the proportion of women with insufficient intakes of two or more micronutrients revealed that 17.5% had insufficient intakes of Vitamin A and iron, 13.7% inadequate intake of iron and zinc, and 43.2% had low intakes of folate and vitamin B12. Mean measured total energy intake was 2196 kcal/day.

**Table 1:** Dietary intakes of selected micronutrients by quintiles of SES among WRA in rural communities in northern Vietnam.

	RDA^1^	EAR^2^^,^ 3	Overall	Lowest	Low	Middle	Higher	Highest^4^	p fordifferences
Iron (mg)	24.5	12.15	15.7^5^	14.4	15.0	15.2	16.2	17.8	<0.0001
			(12.2–20.5)	(11.0–19.0)	(11.9–19.8)	(11.9–19.9)	(12.5–20.5)	(14.0–23.2)	
Zinc (mg)	9.8	7.4	10.0	9.4	9.8	9.9	10.3	10.8	<0.0001
	9.8	7.4	(8.2–12.3)	(7.5–11.5)	(8.1–12.0)	(8.1–12.0)	(8.5–12.5)	(9.0–13.5)	
Vitamin C (mg)	75	60	206	167	192	199	212	265	<0.0001
	75	60	(130–312)	(101–270)	(120–296)	(127–292)	(138–309)	(177–376)	
Vitamin B1 (mg)	1.1	0.9	1.4	1.1	1.2	1.3	1.4	1.6	<0.0001
			(1.0–1.8)	(0.9–1.5)	(1.0–1.7)	(1.0–1.8)	(1.1–1.9)	(1.3–2.2)	
Vitamin B2 (mg)	1.1	0.9	1.0	0.9	1.0	1.0	1.1	1.3	<0.0001
			(0.7–1.5)	(0.6–1.3)	(0.6–1.4)	(0.7–1.4)	(0.7–1.5)	(0.9–1.9)	
Vitamin B3 (mg)	14	11	17.8	15.7	17.1	17.4	18.5	19.9	<0.0001
			(13.7–23.5)	(12.0–21.5)	(13.3–22.7)	(13.5–22.9)	(14.5–23.8)	(16.1–26.7)	
Vitamin B5 (mg)	5	NA	6.9	6.5	6.8	6.8	7.0	7.1	<0.0001
			(5.7–8.3)	(5.3–7.8)	(5.7–8.1)	(5.6–8.2)	(5.9–8.4)	(6.0–8.7)	
Vitamin B6 (mg)	1.3	1.1	1.5	1.3	1.4	1.5	1.6	1.7	<0.0001
			(1.2–1.9)	(1.0–1.7)	(1.1–1.8)	(1.2–1.8)	(1.2–1.9)	(1.3–2.1)	
Folate total (µg)	400	320	304	276	297	290	307	351	<0.0001
			(214–432)	(196–392)	(203–402)	(203–420)	(218–438)	(260–497)	
Vitamin B12 (µg)	2.4	2.0	1.5	1.0	1.2	1.4	1.7	2.2	<0.0001
			(0.8–2.6)	(0.6–2.0)	(0.7–2.2)	(0.8–2.3)	(1.0–2.7)	(1.4–3.6)	
Vitamin A (µg)^6^	600	500	783	679	737	720	781	1007	<0.0001
			(477–1198)	(412–1070)	(439–1144)	(430–1114)	(505–1182)	(653–1442)	
Energy intake (Kcal)			2196±650^7^	2098±668	2157±620	2168±623	2223±632	2341±677	<0.001

1 Recommended dietary allowance for iron is based on assumption of low bioavailability of iron from Vietnamese diet (12%), rather than the 18% from a mixed Western diet. Recommended dietary allowance for zinc also is based on assumption of low bioavailability.

2 Estimated average requirement (EAR) for iron and zinc are also based on assumption of low bioavailability as above.

3 RDA and EAR are based on WHO recommendation for non-pregnant, non-lactating women >19 y [16].

4 Differences across SES groups for all nutrients are statistically significant by Wilcoxon rank test (p<0.0001).

5 Values are median (interquartile range).

6 Vitamin A expressed in retinol activity equivalents (RAE).

7 Values are mean ± SD.

### Food Sources of Key Micronutrients

**Figure 2:**
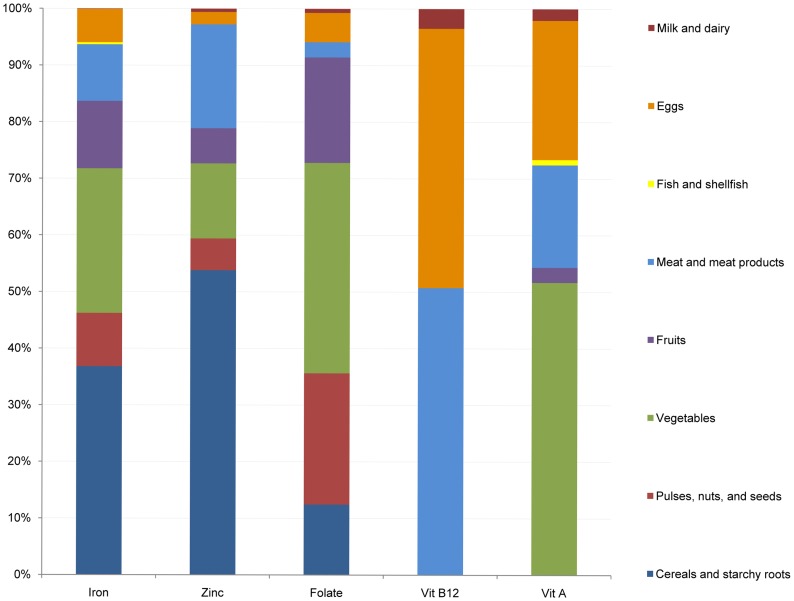
Relative contributions of different food sources to dietary intakes of selected micronutrients for WRA in rural communities in northern Vietnam.

**Figure 3:**
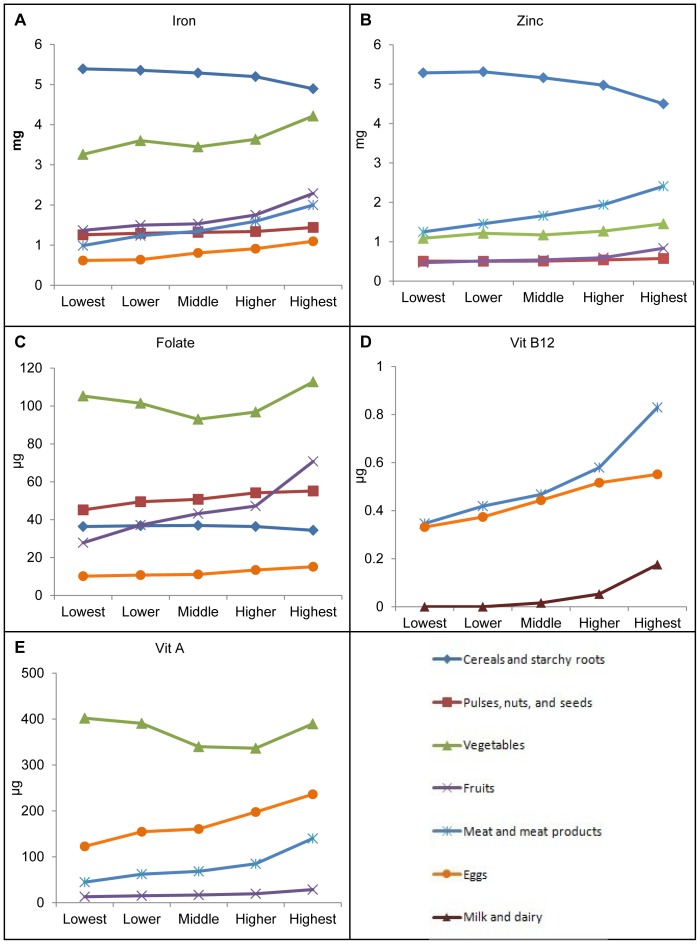
Micronutrient intakes among WRA by food sources and quintiles of SES.

The relative contribution of different food groups to micronutrient intakes are shown in Figure 2. Overall, cereal and starchy foods provided an average of 5.2 g iron (about 37% of total iron). Vegetables provided another 3.6 g iron (25% of total iron). Fruits and nuts provided 1.7 and 1.3 g of iron (around 12% and 9% of total iron), respectively. Only 1.4 g of iron (10%) came from highly bioavailable sources such as meat. The remaining iron intakes were provided by a number of other foods. Specific foods that provided the most iron in this population include rice, chicken, tofu, sauropus leaves, and mustard greens. Iron intake from staple foods did not differ by SES status. However, women with higher SES consumed more iron from vegetables, fruits, meats and eggs (Figure 3-A).

The main sources of dietary zinc were rice, chicken meat, pork, and tofu. Similar to iron, more than half the zinc intake came from staple foods, while meat and meat products provided 18% (1.7 mg/d) and vegetables provided 13% of total zinc intakes (1.2 mg/d) (Figure 2). The contribution from meat, vegetables, and fruits was significantly higher for women from higher SES households compared with those from lower SES (Figure 3-B).

The main dietary sources of folate were vegetables, legumes and fruit (accounting for 35%, 22% and 18% of total folate, respectively) (Figure 2) and included oranges, spinach, peanuts, mustard greens, and rice. Women in higher SES quintiles consumed much more folate from fruit and legume groups (Figure 3-C).

The primary sources of vitamin B12 were meat (51%) and eggs (46%) (Figure 2) with significant differences by quintile of SES. The majority of vitamin B12 was provided by eggs (duck and hen), pork liver and meat, and chicken. Women in the highest wealth quintile consumed nearly three times as much vitamin B12 from meat sources and nearly two times as much vitamin B12 from eggs compared to women in the lowest wealth quintile (Figure 3-D).

The predominant source of vitamin A were vegetables (nearly 50%), followed by eggs (20%) and meat (10%) which contain preformed vitamin A (Figure 2). Specific foods included organ meat (kidney, heart, and liver from pigs), eggs (embryonated duck eggs and chicken eggs), sauropus leaves, spinach, and mustard greens. Women from the lower and higher quintiles of SES were more likely to get vitamin A from vegetables compared to those in the middle, but the consumption of vitamin A from animal sources (meat and eggs) was positively associated with SES (Figure 3-E).

### Determinants of Key Micronutrient Intakes

Bivariate analyses revealed significant positive associations between micronutrient intakes and SES status (Table 1). The greatest differences were for vitamin B12 intakes with a two-fold difference between the highest and lowest quintile of SES, followed by vitamin A, folate and iron. Multivariate analysis revealed that the positive associations between SES and micronutrient intakes remain even after adjusting for age, education, ethnicity, occupation, number of dependents and energy intake (Table 2). When comparing the poorest with the wealthiest quintile, consumption increased 26% for iron (log transformed), 19% for zinc, 36% for folate, 82% for vitamin B12 and 46.8% for vitamin A. The adequacy of intakes also varied significantly by SES for all key nutrients. Multivariate logistic regression models also showed that women in higher SES quintiles were less likely to have insufficient micronutrient intakes when compared to their counterparts in lower SES quintiles (Table 3). Other factors associated with micronutrient intakes include women’s education, ethnicity, and number of dependents in the households (Table 2). Education beyond primary school was associated with higher intakes of iron, folate, vitamin B12 and vitamin A, when compared to those who had not completed primary school. Ethnic minority groups consumed more zinc, folate and vitamin B12 compared to Kinh, but there were no differences for iron and vitamin A. The number of dependents in a household was positively associated with increased micronutrient intakes, after adjusting for other factors.

**Table 2:** Adjusted differences in dietary intakes of selected micronutrients (iron, zinc, folate, vitamin B12 and vitamin A) among WRA in Vietnam based on selected socio-demographic characteristics^1^^,^ 2.

	Iron (mg)	Zinc (mg)	Folate (mg)	Vitamin B12 (mcg)	Vitamin A (RE)
**SES quintile**					
Lowest	1 (Reference)	1 (Reference)	1 (Reference)	1 (Reference)	1 (Reference)
Lower	6.2 (2.5, 9.6)	5.6 (2.6, 8.2)	7.1 (2.2, 11.4)	16.2 (7.7, 22.2)	5.8 (−0.7, 11.9)
Middle	8.3 (10.0, 17.8)	7.5 (4.3, 10.2)	10.5 (5.1, 14.9)	26.2 (15.6, 31.0)	7.5 (0.6, 13.9)
Higher	14.9 (18.3, 27.1)	12.7 (8.9, 15.0)	19.3 (12.6, 22.7)	47.1 (30.6, 46.6)	22.3 (13.3, 27.1)
Highest	25.5 (2.5, 9.6)	19.4 (14.3, 21.2)	35.6 (24.8, 36.2)	81.6 (50.6, 68.6)	47.4 (31.1, 46.6)
**Age (y)** 3	0.3 (0.0, 0.5)	0.0 (−0.2, 0.2)	0.7 (0.4, 1.0)	−0.2 (−0.7, 0.3)	0.7 (0.2, 1.1)
**Education (highest** **grade completed)**					
0–5	1 (Reference)	1 (Reference)	1 (Reference)	1 (Reference)	1 (Reference)
6–9	6.3 (1.9, 10.4)	5.5 (2.1, 8.7)	4.3 (−1.2, 9.7)	30.4 (17.9, 35.2)	14.1 (5.7, 20.7)
10–12	5.8 (0.9, 10.3)	5.3 (1.5, 8.9)	2.4 (−3.8, 8.5)	37.6 (22.3, 41.6)	12.7 (3.6, 20.3)
College or higher	6.1 (−0.1, 12.0)	5.8 (0.9, 10.4)	0.3 (−7.6, 8.2)	37.8 (19.7, 44.5)	10.7 (−0.6, 20.9)
**Ethnicity**					
Kinh	1 (Reference)	1 (Reference)	1 (Reference)	1 (Reference)	1 (Reference)
Minority groups	1.5 (−0.9, 3.8)	3.6 (1.7, 5.4)	3.9 (0.8, 6.9)	7.3 (2.3, 11.9)	0.7 (−3.5, 4.8)
**Occupation**					
Others	1 (Reference)	1 (Reference)	1 (Reference)	1 (Reference)	1 (Reference)
Farmers	−1.5 (−5.5, 2.5)	0.4 (−2.7, 3.5)	−1.7 (−6.8, 3.5)	17.7 (−27.6, −11.3)	−9.6 (−17.1, −3.0)
**Number of dependents**					
0	1 (Reference)	1 (Reference)	1 (Reference)	1 (Reference)	1 (Reference)
1	9.2 (4.7, 13.0)	8.5 (4.9, 11.4)	12.3 (6.2, 17.0)	2.6 (−5.9, 11.1)	16.7 (8.0, 22.8)
> = 2	8.9 (3.7, 13.2)	9.0 (4.9, 12.3)	9.1 (2.5, 14.9)	−1.3 (−11.0, 8.5)	9.4 (0.5, 17.4)

1 Adjusted mean difference (95% Confidence Interval) using multivariate linear regression.

2 Model is also adjusted for energy intake, clustering effects at the commune level and timing of data collection.

3 Values are the coefficient for age as a continuous variable.

**Table 3:** Sociodemographic characteristics associated with the odds of consuming less than estimated average requirement (EAR) of iron, zinc, folate, vitamin B12 and vitamin A among WRA in Vietnam^1^.

	Iron	Zinc	Folate	Vitamin B12	Vitamin A
**SES quintile**					
Lowest	1 (Reference)	1 (Reference)	1 (Reference)	1 (Reference)	1 (Reference)
Lower	0.77 (0.62, 0.95)	0.67 (0.54, 0.83)	0.76 (0.63, 0.92)	0.68 (0.56, 0.82)	0.82 (0.68, 0.99)
Middle	0.69 (0.55, 0.86)	0.66 (0.53, 0.83)	0.67 (0.55, 0.82)	0.65 (0.53, 0.80)	0.82 (0.67, 1.01)
Higher	0.56 (0.45, 0.71)	0.48 (0.37, 0.61)	0.54 (0.44, 0.66)	0.47 (0.38, 0.58)	0.56 (0.45, 0.69)
Highest	0.35 (0.27, 0.45)	0.35 (0.26, 0.47)	0.34 (0.27, 0.43)	0.25 (0.19, 0.32)	0.36 (0.28, 0.46)
**Age (y)**	0.99 (0.97, 1.00)	0.99 (0.98, 1.01)	0.98 (0.97, 1.00)	0.98 (0.97, 0.99)	0.98 (0.97, 1.00)
**Education (highest** **grade completed)**					
0–5	1 (Reference)	1 (Reference)	1 (Reference)	1 (Reference)	1 (Reference)
6–9	0.79 (0.61, 1.01)	0.76 (0.59, 0.98)	0.91 (0.72, 1.13)	0.89 (0.71, 1.11)	0.69 (0.55, 0.87)
10–12	0.90 (0.68, 1.19)	0.76 (0.57, 1.02)	0.97 (0.75, 1.24)	0.81 (0.63, 1.05)	0.73 (0.56, 0.95)
College or higher	0.91 (0.64, 1.29)	0.59 (0.40, 0.89)	1.07 (0.77, 1.48)	0.78 (0.55, 1.10)	0.70 (0.49, 0.99)
**Ethnicity**					
Kinh	1 (Reference)	1 (Reference)	1 (Reference)	1 (Reference)	1 (Reference)
Minority groups	0.92 (0.80, 1.05)	0.90 (0.77, 1.05)	0.89 (0.78, 1.01)	0.89 (0.78, 1.01)	0.96 (0.84, 1.10)
**Occupation**					
Others	1 (Reference)	1 (Reference)	1 (Reference)	1 (Reference)	1 (Reference)
Farmers	1.14 (0.95, 1.47)	0.86 (0.66, 1.12)	1.05 (0.85, 1.30)	1.28 (1.02, 1.62)	1.17 (0.92, 1.48)
**Number of dependents**					
0	1 (Reference)	1 (Reference)	1 (Reference)	1 (Reference)	1 (Reference)
1	0.72 (0.56, 0.93)	0.58 (0.45, 0.74)	0.70 (0.56, 0.87)	0.67 (0.54, 0.84)	0.63 (0.50, 0.79)
> = 2	0.66 (0.50, 0.88)	0.54 (0.40, 0.72)	0.73 (0.57, 0.95)	0.75 (0.58, 0.97)	0.74 (0.57, 0.96)

1 Odds ratio (95% Confidence Interval) using multivariate logistic regression.

## Discussion

This paper is one of the first to describe micronutrient intakes among Vietnamese WRA, based on dietary intake assessment at the individual level. Our findings suggest that micronutrient intakes in WRA from Northern Vietnam are sub-optimal, especially among the poor. This is consistent with results from an analysis of the 2010 Vietnamese Micronutrient Survey where the authors inferred median intakes of iron and vitamin A in WRA were 38% and 61% of the RDA [11]. This insufficient nutrient intake of Vietnamese WRA raises concerns for women’s health both in terms of increased risk of adverse maternal and birth outcomes as well as of long-term consequences related to overall health and wellbeing [5].

Our findings suggest that there is a significant need for establishing healthy eating patterns and improving dietary intakes among all WRA. We find that SES is positively associated with micronutrient intakes (overall and proportion consuming less than EAR) even after adjusting for other socio-demographic characteristics such as age, education and ethnicity. Overall, those with higher SES have more diverse diets compared to the poor who get most of their micronutrient intakes from staples.

Studies have shown that foods such as rice, starchy foods, and other staples that are not rich in micronutrients, and/or have low bioavailability, provide energy at a lower cost than food items rich in micronutrients like beef and poultry [10], [20]. For example, the average cost of providing 1000 calories is only $0.35 for staples in Vietnam compared to $2.12 and $ 3.35 for vegetables and fruits, respectively, and even higher for animal products, namely $2.32, $11.85, and $13.99 for pork, poultry and other meats respectively [10]. These differences explain why poorer households consume a larger amount of staples (77% of calories) compared to wealthier counterparts (59% of calories) [10] to meet their caloric requirements. Targeted interventions that are affordable are therefore needed to help improve the diets of the poor. However, it is noteworthy that diet quality is sub-optimal even among those in the upper quintile of SES. The SES quintiles represent relative wealth among our rural study population, containing more ethnic minorities and poorer households than in urban areas. Data from the Vietnam Household Living Standard Survey showed while only 6.6% of population live below the poverty line in urban areas, 35.6% do so in rural areas [21]. Using the EAR as the cutoff point for inadequate intake, we found that approximately 20–60% of those in the highest wealth quintile had inadequate intakes of iron, vitamin A, B-12 and folate.

A big challenge is the low intake of important sources of bioavailable iron and zinc, such as animal products; which represented only 10% of iron and 18% of zinc intake, respectively. The staples provided the majority of iron and zinc in the Vietnamese diet (contributing to more than a third of iron intake and one half of zinc intake). Micronutrients in these foods are not adequately absorbed, and therefore increasing the consumption of staples is not an effective strategy to prevent deficiencies. Additionally, eating more staple foods might increase calorie and fat intake which could contribute to chronic diseases. Our findings clearly demonstrate the need for strategies that will help families diversify their diets with a balanced variety of foods regardless of their SES status.

A recent systematic review suggested that the promotion of agriculture and small-animal production as potentially promising and culturally relevant strategies to improve and diversify diets [22]. Our findings also show that there are many locally available low-cost food sources that can contribute substantial amounts of micronutrients. Therefore, in order to combat the economic barriers to good nutrition and decrease food insecurity, it is important to encourage local nutrient rich food production and consumption. Around half of vitamin A and folate in participants’ diets came from fruits and vegetables that can be locally produced. Educating and training families in Vietnam on the benefits of planting fruits and vegetables in kitchen and community gardens can empower them to take an active role in their health and reduce the financial burden of having to purchase high cost non-staple foods [23], [24]. Similarly promotion of animal husbandry at the household level could improve the supply of animal source foods that are rich sources of highly bioavailable forms of iron, zinc, and vitamin B12. Encouraging women and communities to raise livestock will be an important strategy to help combat micronutrient deficiency. Expanding poverty and malnutrition reduction programs like the Centre of Training and Performing Techniques, which promotes the improvement of veterinary services and breeding, and the Vuon Ao Chuong (VAC, *Garden Pond Cattle Shed*), an institution that provides education and resources to address nutritional demands, is also recommended [23]. Initiatives such as the nutrition education and rehabilitation programs of Save the Children that include community empowerment and nutrition are an option to improve food choices, meal planning, feeding practices, hygiene, and food preparation in Vietnam through culturally acceptable and financially feasible methods [25].

Micronutrient supplementation and food fortification are recommended in the Millennium Development Goals to eradicate poverty, decrease the rate of child mortality, and improve maternal health status [22], and are under consideration by the Vietnamese government in the Strategy for Prevention and Control of Micronutrient Deficiencies in Vietnam 2001–2010. In our study, rice and other grains are the main foods, contributing for 65.6% of energy intake [26], making rice the best candidate for food fortification. Findings from a recent study showed that fortifying rice could increase iron intake by 41.4% of the recommended nutrient intake, zinc by 15.5% and folate by 34.1% [11]. However, the consumption of fortified foods is confined largely to urban centers and many rural communities that typically rely on their own production may have limited access to fortified food products. Therefore, the promotion of food based approaches, including improving agricultural practices and/or diversifying food production combines with promoting better dietary practices, is appropriate and needed [23].

Important strengths of this work are the large sample size and use of a validated semi-quantitative food frequency questionnaire that included 107 common food and beverage items to calculate nutrient intakes. The FFQ is the most commonly used and recommended method to determine and evaluate micronutrient intakes in large epidemiological studies [27], [28]. The main advantages are low cost and ability to characterize the usual diet in the past as well as to minimize the risk of interviewer and measurement bias. The drawbacks of the FFQ include the use of fixed lists of foods, the reliance of accuracy of recall and the difficulties in portion size estimation. We overcame these problems by asking a comprehensive validated 107 item food list and using standardized set of tableware with a recall period during the last 3 months. Micronutrient intakes can also be affected by seasonality. Our data were collected in the winter/spring when vegetables are available but the availability of fruits is less compared to other seasons [29], [30] which may have resulted in systematic biases in our estimates of the intakes of nutrients such as vitamin A and folate. Participants in this study were WRA from a specific province of Vietnam, and thus do not necessarily represent the entire Vietnamese population. Nonetheless, we included a large sample, all women in the community who met the inclusion criteria had equal opportunity to participate, and there were no differences in baseline characteristics between participants and non-participants.

In conclusion, findings from this study provide valuable information that can be used to design better policies and intervention programs to help improve nutritional choices, increase micronutrient intakes and improve overall health among WRA in Vietnam. Our study has provided specific details on the local foods supplying the majority of micronutrients to WRA in all wealth quintiles. This new understanding of micronutrient intake allows us to tailor our recommendations for nutrition interventions that promote local and sustainable food sources. Targeted efforts to promote the consumption of local nutrient rich foods along with educational programs and social development are needed in order to improve micronutrient and health status of women in Vietnam.

## References

[1] Bhutta ZA, Gupta I, de’Silva H, Manandhar D, Awasthi S, et al (2004) Maternal and child health: is South Asia ready for change? BMJ 328: 816–819. 15070640 10.1136/bmj.328.7443.816 PMC383381

[2] Seshadri S (2001) Prevalence of micronutrient deficiency particularly of iron, zinc and folic acid in pregnant women in South East Asia. Br J Nutr 85 Suppl 2S87–92. 11509095

[3] WHO (2012) World Health Organization. Nutrition: Micronutrients. Retrieved from http://www.who.int/nutrition/topics/micronutrients/en/.

[4] Ramakrishnan U, Huffman S (2008) Multiple Micronutrient Malnutrition: What Can Be Done? In: Nutrition and Health in Developing Countries. Ed: Semba, R.D., Bloem, M. Totawa, NJ: Humana Press.

[5] Ramakrishnan U, Grant F, Goldenberg T, Zongrone A, Martorell R (2012) Effect of Women’s Nutrition before and during Early Pregnancy on Maternal and Infant Outcomes: A Systematic Review Paediatric and Perinatal Epidemiology. 26: 285–301. 10.1111/j.1365-3016.2012.01281.x 22742616

[6] Huy ND, Le Hop T, Shrimpton R, Hoa CV (2009) An effectiveness trial of multiple micronutrient supplementation during pregnancy in Vietnam: impact on birthweight and on stunting in children at around 2 years of age. Food Nutr Bull 30: S506–516. 20120792 10.1177/15648265090304S405

[7] NIN MOH, UNICEF (2012) Summary Report General Nutrition Survey 2009–2010. http://www.unicef.org/vietnam/resources_18459.html.

[8] Laillou A, Pham TV, Tran NT, Le HT, Wieringa F, et al (2012) Micronutrient deficits are still public health issues among women and young children in Vietnam. PLoS One 7: e34906. 22529954 10.1371/journal.pone.0034906 PMC3328495

[9] Dien le N, Thang NM, Bentley ME (2004) Food consumption patterns in the economic transition in Vietnam. Asia Pac J Clin Nutr 13: 40–47. 15003913

[10] Hoang LV (2009) Analysis of Calorie and Micronutrient Consumption in Vietnam. DEPOCEN Working Paper Series, Center for Agricultural Policy, Institute of Policy and Strategy for Agriculture and Rural Development.

[11] Laillou A, Berger J, Le BM, Pham VT, Le TH, et al (2012) Improvement of the Vietnamese diet for women of reproductive age by micronutrient fortification of staples foods and condiments. PLoS One 7: e50538. 23226308 10.1371/journal.pone.0050538 PMC3511532

[12] Nguyen PH, Lowe AE, Martorell R, Nguyen H, Pham H, et al (2012) Rationale, design, methodology and sample characteristics for the Vietnam pre-conceptual micronutrient supplementation trial (PRECONCEPT): a randomized controlled study. BMC Public Health 12: 898. 23092451 10.1186/1471-2458-12-898 PMC3533960

[13] Tran DT (2009) FFQ for adult in rural Vietnam. In “An examination of the relationship between low body mass index and micronutrient malnutrition and the risk of morbidity in adults aged 18 to 60 in rural Vietnam”, PhD thesis, The University of Newcastle, Australia.

[14] NIN MOH (2007) Vietnamese Food Composition Table. Hanoi: Medical Publishing Housing.

[15] Yen H (2009) 555 Vietnamese Dishes -Cooking Technique and Nutrient Contents. Hanoi: Tu dien Bach khoa Publishing House.

[16] WHO FAO (2004) Vitamin and mineral requirements in human nutrition. World Health Organization and Food and Agriculture Organization of the United Nations. Geneva, Switzerland.

[17] Vyas S, Kumaranayake L (2006) Constructing socio-economic status indices: how to use principal components analysis. Health Policy Plan 21: 459–468. 17030551 10.1093/heapol/czl029

[18] Gwatkin D, Rutstein S, Johnson K, Suliman E, Wagstaff A, et al (2007) Socio-economic differences in health, nutrition, and population within developing countries: an overview. Niger J Clin Pract 10: 272–282. 18293634

[19] SAS (2004) SAS, the power to know. SAS Institue Inc.; 2002–2004.

[20] Molini V (2006) Food Security in Vietnam during the 1990s. The Empirical Evidence. Research Paper No. 2006/67. Center for World Food Studies.

[21] Swinkels R, Turk C (2004) Poverty and remote areas: evidence from new data and questions for the future. Background paper for the PAC conference, 24–26 November 2004 World Bank, Vietnam.

[22] Bhutta ZA, Ahmed T, Black RE, Cousens S, Dewey K, et al (2008) What works? Interventions for maternal and child undernutrition and survival. Lancet 371: 417–440. 18206226 10.1016/S0140-6736(07)61693-6

[23] Hop le T (2003) Programs to improve production and consumption of animal source foods and malnutrition in Vietnam. J Nutr 133: 4006S–4009S. 14672303 10.1093/jn/133.11.4006S

[24] Hop Le T, Van TK, Thanh HK (2011) Food based dietary guidelines in Vietnam: progress and lessons learned. Asia Pac J Clin Nutr 20: 495–499. 21859672

[25] Dickey VC, Pachon H, Marsh DR, Lang TT, Claussenius DR, et al (2002) Implementation of nutrition education and rehabilitation programs (NERPs) in Viet Nam. Food Nutr Bull 23: 78–85. 12503235

[26] Nguyen PH, Strizich G, Lowe A, Nguyen H, Pham H, et al (2013) Food consumption patterns and associated factors among Vietnamese women of reproductive age. Nutr J 12: 126. 24028650 10.1186/1475-2891-12-126 PMC3847174

[27] Henriquez-Sanchez P, Sanchez-Villegas A, Doreste-Alonso J, Ortiz-Andrellucchi A, Pfrimer K, et al (2009) Dietary assessment methods for micronutrient intake: a systematic review on vitamins. Br J Nutr 102 Suppl 1S10–37. 20100364 10.1017/S0007114509993126

[28] Serra-Majem L, Pfrimer K, Doreste-Alonso J, Ribas-Barba L, Sanchez-Villegas A, et al (2009) Dietary assessment methods for intakes of iron, calcium, selenium, zinc and iodine. Br J Nutr 102 Suppl 1S38–55. 20100367 10.1017/S0007114509993138

[29] IFPRI (2002) Fruits and Vegetables in Vietnam: Adding Value from Farmer to Consumer. International Food Policy Research Institute.

[30] FAVRI (2010) Vegetable market reserach in Vietnam. Hanoi, Vietnam: Fruit and Vegetable Research Institute, Viet Nam in collaboration with FAO.

